# A ramsayite-type oxide, Ca_2_Sn_2_Al_2_O_9_

**DOI:** 10.1107/S1600536810036445

**Published:** 2010-09-18

**Authors:** Hisanori Yamane, Shunsuke Abe, Rong Tu, Takashi Goto

**Affiliations:** a Institute of Multidisciplinary Research for Advanced Materials, Tohoku University, 2-1-1 Katahira, Aoba-ku, Sendai 980-8577, Japan; b Institute for Materials Research, Tohoku University, 2-1-1 Katahira, Aoba-ku, Sendai 980-8577, Japan

## Abstract

The title compound, dicalcium nonaoxidodistannate(IV)dialuminate, is the second example which crystallizes in the isotypic structure of a pyroxene silicate, Na_2_Ti_2_Si_2_O_9_ (ramsayite). _∞_^1^[Sn_2_O_8_] chains and pyroxene-type _∞_^1^[Al_2_O_6_] chains are formed along the *b* axis by sharing O atoms. The Ca atoms are situated in the resulting channels and exhibit a coordination number of 7.

## Related literature

For the structure of ramsayite, Na_2_Ti_2_Si_2_O_9_, see: Sundberg *et al.* (1987 ▶). For the synthesis of Ca_8_Sn_7_Al_10_O_37_, see: Barbanyagre & Kotlyarov (2001 ▶). For the structure of a related stannate silicate, Ca_2_SnSi_2_O_9_, see: Blasse *et al.* (1995 ▶). For bond-valence parameters, see: Brese & O’Keeffe (1991 ▶). For the CaO–Al_2_O_3_ system, see: Jerebtsov & Mikhailov (2001 ▶). For the Inorganic Crystal Structure Database, see: ICSD (2009 ▶).

## Experimental

### 

#### Crystal data

Ca_2_Sn_2_Al_2_O_9_
*M* *_r_* = 515.50
Orthorhombic, 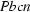

*a* = 8.9866 (6) Å
*b* = 5.4894 (11) Å
*c* = 14.9030 (18) Å
*V* = 735.18 (18) Å^3^
*Z* = 4
Mo *K*α radiation
μ = 8.46 mm^−1^
*T* = 293 K
0.17 × 0.15 × 0.07 mm

#### Data collection

Rigaku R-AXIS RAPID diffractometer
Absorption correction: numerical (*NUMABS*; Higashi, 1999 ▶) *T*_min_ = 0.427, *T*_max_ = 0.717
6534 measured reflections
845 independent reflections
788 reflections with *I* > 2σ(*I*)
*R*_int_ = 0.051

#### Refinement

*R*[*F* ^2^ > 2σ(*F* ^2^)] = 0.018
*wR*(*F* ^2^) = 0.043
*S* = 1.08
845 reflections
69 parameters
Δρ_max_ = 0.72 e Å^−3^
Δρ_min_ = −0.72 e Å^−3^

### 

Data collection: *PROCESS-AUTO* (Rigaku, 1998 ▶); cell refinement: *PROCESS-AUTO*; data reduction: *CrystalStructure* (Rigaku, 2005 ▶); program(s) used to solve structure: *SIR2004* (Burla *et al.*, 2005 ▶); program(s) used to refine structure: *SHELXL97* (Sheldrick, 2008 ▶); molecular graphics: *VESTA* (Momma & Izumi, 2008 ▶); software used to prepare material for publication: *SHELXL97*.

## Supplementary Material

Crystal structure: contains datablocks I, global. DOI: 10.1107/S1600536810036445/br2147sup1.cif

Structure factors: contains datablocks I. DOI: 10.1107/S1600536810036445/br2147Isup2.hkl

Additional supplementary materials:  crystallographic information; 3D view; checkCIF report

## Figures and Tables

**Table 1:** Selected bond lengths (Å)

Ca1—O3^i^	2.303 (2)
Ca1—O2	2.391 (2)
Ca1—O1^ii^	2.404 (2)
Ca1—O4^i^	2.412 (2)
Ca1—O2^i^	2.463 (2)
Ca1—O5	2.486 (2)
Ca1—O3^iii^	2.624 (2)
Al1—O2^i^	1.735 (2)
Al1—O4	1.745 (2)
Al1—O2	1.763 (2)
Al1—O1^iv^	1.777 (3)
Sn1—O4^v^	2.002 (2)
Sn1—O3	2.032 (2)
Sn1—O3^i^	2.053 (2)
Sn1—O5^vi^	2.0682 (13)
Sn1—O1^i^	2.106 (2)
Sn1—O1	2.207 (2)

Symmetry codes: (i) 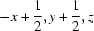; (ii) 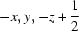; (iii) 
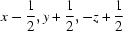
; (iv) 
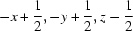
; (v) 
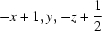
; (vi) 
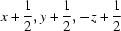
.

**Fig. 1.**
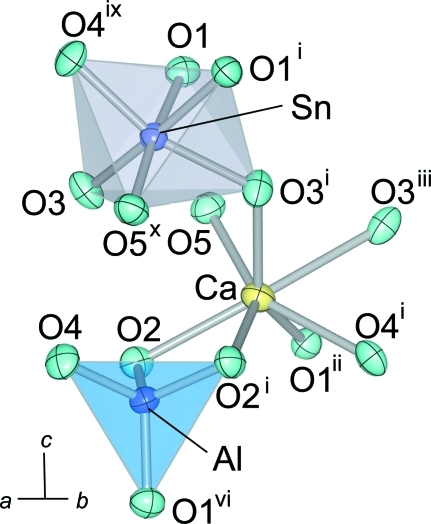
O-atom coordination around Ca, Al, and Sn atoms in the structure of Ca2Sn2Al2O9. Displacement ellipsoids are drawn at 99% probability level. [Symmetry codes as in Table 1.]

**Fig. 2.**
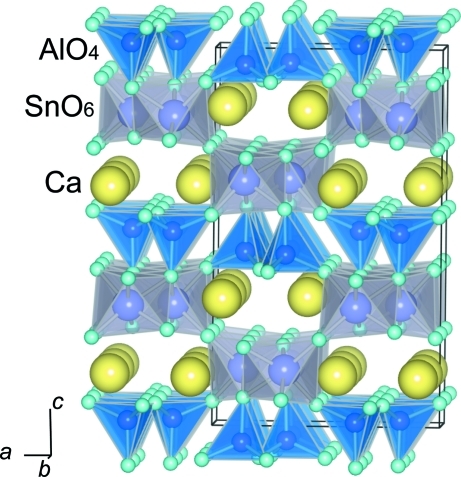
Crystal structure of Ca2Sn2Al2O9 illustrated with distorted Sn-centered oxygen octahedra and Al-centered oxygen tetrahedra.

## supplementary crystallographic information

### Comment

Barbanyagre and Kotlyarov (2001) reported a quaternary oxide Ca_8_Al_10_Sn_7_O_37_ with a powder X-ray diffraction (PXRD) pattern, but they did not clarify the crystal structure. We have prepared single crystals of Ca_2_Sn_2_Al_2_O_9_ by slow cooling of a Ca—Al—Sn—O melt. The PXRD pattern of Ca_8_Al_10_Sn_7_O_37_ was similar to that calculated with the crystal structure parameters of Ca_2_Sn_2_Al_2_O_9_ analyzed by the present study.

Ca_2_Sn_2_Al_2_O_9_ is isostructural with ramsayite (lorenzenite) Na_2_Ti_2_Si_2_O_9_ (*a* = 8.7128 (10), *b* = 5.2327 (5), *c* = 14.487 (2) Å) (Sundberg, *et al.*, 1987). Other compounds crystallizing in the isotypic structure were not found in the Inorganic Crystal Structure Database (ICSD, 2009).

The selected bond distances in Ca_2_Sn_2_Al_2_O_9_ are summarized in Table 1. The coordination environments of Ca, Sn and Al are drawn with displacement ellipsoids in Fig. 1. The Sn site is in a distorted oxygen octhahedron with the Sn—O distances varying from 2.002 (2) to 2.207 (2) Å, (average 2.08 (8) Å). The bond valence sum (BVS) calculated for Sn atoms with the bond valence parameter of *R* (Sn—O) = 1.905 Å (Brese & O'Keeffe, 1991) was 3.815, a little smaller than the IV valence of Sn. These Sn—O bond lengths and the BVS are consistent with those reported for Ca_3_SnSi_2_O_9_ (2.007–2.134 Å, avg. 2.08 (4) Å, BVS 3.803) (Blasse *et al.*, 1995).

Each SnO_6_ octahedron shares O1—O3 edges with both adjacent sides of SnO_6_ octahedra and a one-dimensional chain _∞_^1^[Sn_2_O_8_] is formed along the *b* axis. Al atoms are tetrahedrally coordinated by O atoms and the AlO_4_ tetrahedra form pyroxene-type _∞_^1^[Ali_2_O_6_] chains along the *b* axis by sharing O2. The Al—O bond lengths are from 1.735 to 1.777 (3) Å (avg. 1.755 (19) Å) and the BVS of 3.02 is consistent with trivalent of Al(III). The chains of _∞_^1^[Ali_2_O_6_] and _∞_^1^[Sn_2_O_8_] are bridged by sharing O4, and the _∞_^1^[Sn_2_O_8_] chains are linked each other by sharing O5 on a two-fold axis, forming rectangular channels along the *b* axis (Fig. 2). Ca sites are situated in the channels and surrounded by 7 oxygen atoms with the distances from 2.303 (2) to 2.624 (2) Å. The BVS of Ca atoms is 2.01 and well agrees with the valence number of Ca(II).

A permittivity of 48 and a tan*δ* of 0.02 were measured at room temperature with an impedance analyzer (Solartron 1260 and 1296) for the polycrystalline sample which was mainly composed of Ca_2_Sn_2_Al_2_O_9_ with a small amount of CaSnO_3_ which crystallized at the initial stage of solid state reaction.

### Experimental

The starting materials used were CaCO_3_ (Rare Metallic, 99.99% purity), SnO_2_ (Rare Metallic, 99.99% purity) and Al_2_O_3_ (Rare Metallic, 99.99% purity). The powders were weighed, mixed in an agate mortar with a pestle, and pressed into pellets, which were placed on a platinum plate and heated in an electric furnace in air. Single crystals of Ca_2_Sn_2_Al_2_O_9_ were prepared from a starting mixture with an atomic ratio Ca: Sn: Al ═ 5:1:4. The pellet of the mixture was heated to 1823 K at a heating rate of 200 K/h, and then cooled to 1773 K at a cooling rate of 5 K/h. The single crystals were probably grown in a flux with a composition close to a Ca_3_Al_2_O_6_—CaAl_2_O_4_ mixture which has the eutectic point of 1644 K (Jerebtsov & Mikhailov, 2001). The samples were then cooled in the furnace by shutting off the electric power. The obtained sample was crushed into fragments and colorless transparent single crystals of about 0.06–0.14 mm were picked up under an optical microscope. A polycrystalline sample was prepared by heating the pellets of the starting mixtures with stoichiometric metal ratios Ca:Sn:Al ═ 1:1:1 at around 1600 K for 24 h.

### Refinement

The highest peak in the difference electron density map is 0.94 Å from Sn1 while the deepest hole is 1.08 Å from the same atom.

### Crystal data

**Table:**

Ca_2_Sn_2_Al_2_O_9_	*F*(000) = 952
*M**_r_* = 515.50	*D*_x_ = 4.657 Mg m^−^^3^
Orthorhombic, *P**b**c**n*	Mo *K*α radiation, λ = 0.71075 Å
Hall symbol: -P 2n 2ab	Cell parameters from 5414 reflections
*a* = 8.9866 (6) Å	θ = 3.6–27.8°
*b* = 5.4894 (11) Å	µ = 8.46 mm^−^^1^
*c* = 14.9030 (18) Å	*T* = 293 K
*V* = 735.18 (18) Å^3^	Platelet, colourless
*Z* = 4	0.17 × 0.15 × 0.07 mm

### Data collection

**Table:**

Rigaku R-AXIS RAPID diffractometer	845 independent reflections
Radiation source: fine-focus sealed tube	788 reflections with *I* > 2σ(*I*)
graphite	*R*_int_ = 0.051
Detector resolution: 10.00 pixels mm^-1^	θ_max_ = 27.5°, θ_min_ = 3.6°
ω scans	*h* = −11→11
Absorption correction: numerical (*NUMABS*; Higashi, 1999)	*k* = −7→7
*T*_min_ = 0.427, *T*_max_ = 0.717	*l* = −19→19
6534 measured reflections	

### Refinement

**Table:**

Refinement on *F*^2^	0 restraints
Least-squares matrix: full	Primary atom site location: structure-invariant direct methods
*R*[*F*^2^ > 2σ(*F*^2^)] = 0.018	Secondary atom site location: difference Fourier map
*wR*(*F*^2^) = 0.043	*w* = 1/[σ^2^(*F*_o_^2^) + (0.002*P*)^2^ + 2.2176*P*] where *P* = (*F*_o_^2^ + 2*F*_c_^2^)/3
*S* = 1.08	(Δ/σ)_max_ = 0.001
845 reflections	Δρ_max_ = 0.72 e Å^−^^3^
69 parameters	Δρ_min_ = −0.72 e Å^−^^3^

### Special details

**Table:**

Geometry. All e.s.d.'s (except the e.s.d. in the dihedral angle between two l.s. planes) are estimated using the full covariance matrix. The cell e.s.d.'s are taken into account individually in the estimation of e.s.d.'s in distances, angles and torsion angles; correlations between e.s.d.'s in cell parameters are only used when they are defined by crystal symmetry. An approximate (isotropic) treatment of cell e.s.d.'s is used for estimating e.s.d.'s involving l.s. planes.
Refinement. Refinement of *F*^2^ against ALL reflections. The weighted *R*-factor *wR* and goodness of fit *S* are based on *F*^2^, conventional *R*-factors *R* are based on *F*, with *F* set to zero for negative *F*^2^. The threshold expression of *F*^2^ > σ(*F*^2^) is used only for calculating *R*-factors(gt) *etc*. and is not relevant to the choice of reflections for refinement. *R*-factors based on *F*^2^ are statistically about twice as large as those based on *F*, and *R*- factors based on ALL data will be even larger.

### Fractional atomic coordinates and isotropic or equivalent isotropic displacement parameters (Å^2^)

**Table:**

	*x*	*y*	*z*	*U*_iso_*/*U*_eq_	
Ca1	0.06714 (7)	0.36160 (11)	0.14603 (5)	0.00676 (14)	
Al1	0.34369 (10)	0.31037 (18)	0.02817 (7)	0.0055 (2)	
Sn1	0.34459 (2)	0.36611 (4)	0.333981 (15)	0.00525 (9)	
O1	0.1618 (2)	0.1912 (4)	0.40897 (16)	0.0070 (5)	
O2	0.2277 (2)	0.0795 (4)	0.07144 (15)	0.0071 (4)	
O3	0.3314 (2)	0.0365 (4)	0.27250 (16)	0.0079 (5)	
O4	0.5156 (2)	0.2310 (4)	0.07402 (16)	0.0078 (4)	
O5	0.0000	0.0250 (6)	0.2500	0.0077 (6)	

### Atomic displacement parameters (Å^2^)

**Table:**

	*U*^11^	*U*^22^	*U*^33^	*U*^12^	*U*^13^	*U*^23^
Ca1	0.0069 (3)	0.0071 (3)	0.0062 (3)	−0.0007 (2)	0.0006 (2)	−0.0003 (2)
Al1	0.0057 (5)	0.0058 (5)	0.0050 (5)	−0.0001 (3)	−0.0004 (3)	0.0001 (4)
Sn1	0.00529 (13)	0.00501 (14)	0.00544 (14)	0.00004 (7)	−0.00022 (7)	−0.00022 (8)
O1	0.0080 (11)	0.0067 (11)	0.0064 (12)	−0.0008 (8)	−0.0004 (8)	−0.0002 (9)
O2	0.0089 (11)	0.0054 (10)	0.0070 (11)	−0.0007 (8)	−0.0005 (9)	−0.0003 (9)
O3	0.0077 (11)	0.0064 (11)	0.0096 (13)	−0.0008 (8)	0.0026 (9)	−0.0019 (9)
O4	0.0059 (10)	0.0088 (11)	0.0087 (11)	−0.0003 (8)	−0.0024 (9)	−0.0011 (9)
O5	0.0086 (15)	0.0078 (15)	0.0066 (16)	0.000	−0.0014 (13)	0.000

### Geometric parameters (Å, °)

**Table:**

Ca1—O3^i^	2.303 (2)	Sn1—O1^i^	2.106 (2)
Ca1—O2	2.391 (2)	Sn1—O1	2.207 (2)
Ca1—O1^ii^	2.404 (2)	O1—Al1^vii^	1.777 (3)
Ca1—O4^i^	2.412 (2)	O1—Sn1^viii^	2.106 (2)
Ca1—O2^i^	2.463 (2)	O1—Ca1^ii^	2.404 (2)
Ca1—O5	2.486 (2)	O2—Al1^viii^	1.735 (2)
Ca1—O3^iii^	2.624 (2)	O2—Ca1^viii^	2.463 (2)
Al1—O2^i^	1.735 (2)	O3—Sn1^viii^	2.053 (2)
Al1—O4	1.745 (2)	O3—Ca1^viii^	2.303 (2)
Al1—O2	1.763 (2)	O3—Ca1^ix^	2.624 (2)
Al1—O1^iv^	1.777 (3)	O4—Sn1^v^	2.002 (2)
Sn1—O4^v^	2.002 (2)	O4—Ca1^viii^	2.412 (2)
Sn1—O3	2.032 (2)	O5—Sn1^x^	2.0682 (13)
Sn1—O3^i^	2.053 (2)	O5—Sn1^viii^	2.0682 (13)
Sn1—O5^vi^	2.0682 (13)	O5—Ca1^ii^	2.486 (2)
			
O3^i^—Ca1—O2	114.30 (8)	O3^i^—Sn1—O1^i^	80.21 (9)
O3^i^—Ca1—O1^ii^	141.26 (8)	O5^vi^—Sn1—O1^i^	88.98 (9)
O2—Ca1—O1^ii^	96.08 (8)	O4^v^—Sn1—O1	87.66 (9)
O3^i^—Ca1—O4^i^	97.80 (8)	O3—Sn1—O1	78.32 (9)
O2—Ca1—O4^i^	121.59 (8)	O3^i^—Sn1—O1	81.41 (9)
O1^ii^—Ca1—O4^i^	84.93 (8)	O5^vi^—Sn1—O1	173.05 (6)
O3^i^—Ca1—O2^i^	82.57 (8)	O1^i^—Sn1—O1	94.57 (9)
O2—Ca1—O2^i^	69.69 (4)	Al1^vii^—O1—Sn1^viii^	121.70 (13)
O1^ii^—Ca1—O2^i^	132.50 (8)	Al1^vii^—O1—Sn1	121.93 (11)
O4^i^—Ca1—O2^i^	67.76 (7)	Sn1^viii^—O1—Sn1	96.90 (9)
O3^i^—Ca1—O5	84.02 (7)	Al1^vii^—O1—Ca1^ii^	108.61 (10)
O2—Ca1—O5	87.41 (7)	Sn1^viii^—O1—Ca1^ii^	97.21 (9)
O1^ii^—Ca1—O5	73.48 (6)	Sn1—O1—Ca1^ii^	107.17 (10)
O4^i^—Ca1—O5	145.85 (7)	Al1^viii^—O2—Al1	134.02 (15)
O2^i^—Ca1—O5	145.50 (6)	Al1^viii^—O2—Ca1	120.07 (11)
O3^i^—Ca1—O3^iii^	77.78 (9)	Al1—O2—Ca1	93.51 (9)
O2—Ca1—O3^iii^	159.97 (8)	Al1^viii^—O2—Ca1^viii^	91.78 (9)
O1^ii^—Ca1—O3^iii^	67.00 (8)	Al1—O2—Ca1^viii^	94.11 (10)
O4^i^—Ca1—O3^iii^	69.47 (7)	Ca1—O2—Ca1^viii^	123.82 (10)
O2^i^—Ca1—O3^iii^	129.46 (7)	Sn1—O3—Sn1^viii^	104.43 (10)
O5—Ca1—O3^iii^	77.73 (6)	Sn1—O3—Ca1^viii^	135.82 (11)
O2^i^—Al1—O4	113.24 (12)	Sn1^viii^—O3—Ca1^viii^	118.71 (10)
O2^i^—Al1—O2	104.92 (9)	Sn1—O3—Ca1^ix^	94.02 (9)
O4—Al1—O2	101.59 (11)	Sn1^viii^—O3—Ca1^ix^	104.41 (10)
O2^i^—Al1—O1^iv^	111.43 (12)	Ca1^viii^—O3—Ca1^ix^	84.64 (7)
O4—Al1—O1^iv^	114.50 (11)	Al1—O4—Sn1^v^	137.01 (13)
O2—Al1—O1^iv^	110.23 (11)	Al1—O4—Ca1^viii^	96.38 (10)
O4^v^—Sn1—O3	90.89 (9)	Sn1^v^—O4—Ca1^viii^	101.55 (9)
O4^v^—Sn1—O3^i^	163.28 (9)	Sn1^x^—O5—Sn1^viii^	130.13 (15)
O3—Sn1—O3^i^	99.18 (9)	Sn1^x^—O5—Ca1^ii^	121.78 (5)
O4^v^—Sn1—O5^vi^	98.44 (7)	Sn1^viii^—O5—Ca1^ii^	95.75 (4)
O3—Sn1—O5^vi^	98.16 (9)	Sn1^x^—O5—Ca1	95.75 (4)
O3^i^—Sn1—O5^vi^	93.33 (8)	Sn1^viii^—O5—Ca1	121.78 (5)
O4^v^—Sn1—O1^i^	88.14 (9)	Ca1^ii^—O5—Ca1	83.96 (10)
O3—Sn1—O1^i^	172.86 (9)		

Symmetry codes: (i) −*x*+1/2, *y*+1/2, *z*; (ii) −*x*, *y*, −*z*+1/2; (iii) *x*−1/2, *y*+1/2, −*z*+1/2; (iv) −*x*+1/2, −*y*+1/2, *z*−1/2; (v) −*x*+1, *y*, −*z*+1/2; (vi) *x*+1/2, *y*+1/2, −*z*+1/2; (vii) −*x*+1/2, −*y*+1/2, *z*+1/2; (viii) −*x*+1/2, *y*−1/2, *z*; (ix) *x*+1/2, *y*−1/2, −*z*+1/2; (x) *x*−1/2, *y*−1/2, −*z*+1/2.
